# A 90.9 dB SNDR 95.3 dB DR Audio Delta–Sigma Modulator with FIA-Assisted OTA

**DOI:** 10.3390/s24051449

**Published:** 2024-02-23

**Authors:** Gongxing Huang, Cong Wei, Rongshan Wei

**Affiliations:** College of Physics and Information Engineering, Fuzhou University, Fuzhou 350116, China

**Keywords:** delta–sigma modulator, analog-to-digital conversion, discrete-time, floating inverter–amplifier, audio

## Abstract

This paper presents a low-power, high-gain integrator design that uses a cascode operational transconductance amplifier (OTA) with floating inverter–amplifier (FIA) assistance. Compared to a traditional cascode, the proposed integrator can achieve a gain of 80 dB, while reducing power consumption by 30%. Upon completing the analysis, the value of the FIA drive capacitor and clock scheme for the FIA-assisted OTA were obtained. To enhance the dynamic range (DR) and mitigate quantization noise, a tri-level quantizer was employed. The design of the feedback digital-to-analog converter (DAC) was simplified, as it does not use additional mismatch shaping techniques. A third-order, discrete-time delta–sigma modulator was designed and fabricated in a 0.18 μm complementary metal-oxide semiconductor (CMOS) process. It operated on a 1.8 V supply, consuming 221 µW with a 24 kHz bandwidth. The measured SNDR and DR were 90.9 dB and 95.3 dB, respectively.

## 1. Introduction

High-precision delta–sigma modulators (DSMs) are widely used to process narrow-band signals, with frequencies ranging from direct current to several kHz. The growing demand for mobile devices, coupled with the evolution of wireless devices, is driving the advancement of portable energy and low-power systems. Discrete-time DSMs (DTDSMs) composed of switched-capacitor (SC) circuits are more prevalent in the industry compared to continuous-time DSMs. This is because of the advantages of better system matching, robustness to process, voltage, and temperature (PVT) variations, and insensitivity to clock jitter [1,2,3,4]. However, the sampling and integration of a DTDSM must be settled within half a sampling clock period. This requirement, that the bandwidth of the OTA must exceed the sampling frequency by several times [5], poses a significant challenge to the development of low-power DTDSMs. In a typical DTDSM, the operational transconductance amplifier (OTA) is often the module that consumes the most power due to its requirement for a large static bias current [6]. However, during the sampling phase, this static bias current leads to a significant power loss. This suggests that conventional high-gain cascode OTAs offer no advantage in the design of low-power DTDSMs. Consequently, finding an alternative to these conventional OTAs for greater energy efficiency presents a considerable challenge.

In an ideal SC integrator, the integration function mimics an exponential curve, meaning the requisite current for integration decreases over time. However, the limited bandwidth of conventional OTAs transforms the integration process into a partial slewing process. This results in the distortion of the integrator output and reduced linearity. A common method to ensure a stable settling time involves sacrificing static power consumption [7]. However, the demand for the output current of the SC integrator decreases with time, and a large portion of the power consumption is wasted. The inverter, a simple OTA, is ideally suited for SC integrators due to its driving capability, which varies with the input. Moreover, at low power voltage, the inverter operates in the weak inversion region, resulting in superior energy efficiency [8,9,10,11]. However, the gain of the inverter is relatively low, typically around 30 dB, which limits the system’s accuracy. To address this limitation, the cascade inverter [12,13] has been proposed. It can achieve a gain of approximately 70 dB for the class-AB OTA, thereby overcoming the issue of insufficient gain of the inverter. However, the self-bias of the inverter still experiences power loss during the sampling phase of the integrator and requires a large capacitor to store the bias voltage. The floating inverter–amplifier (FIA) [14] structure was proposed for use as a pre-amplifier for the comparator. This amplifier was soon used in DTDSM circuits. The FIA, functioning as a full dynamic OTA, is an energy-efficient structure that avoids static current consumption during the bias phase. Additionally, its capacitor-powered mechanism eliminates the requirement for additional common-mode feedback circuitry [15,16]. To compensate for the FIA’s insufficient gain, the high-gain cascade FIA [17,18] structure was proposed. Nevertheless, the gain sensitivity of the inverter structure to PVT variations remains a consideration.

This paper proposes a high-gain FIA-assisted OTA (FAO) to address the problems of the low energy efficiency of conventional cascade OTAs and the low gain of the inverter structure. The FAO can achieve a low-power, high-gain integrator. The paper presents the implementation of an FAO and provides a detailed analysis of its performance and robustness. A tri-level quantizer was used to reduce the quantization noise of the system without additional mismatch shaping circuits. The rest of this article is organized as follows. Section 2 analyzes the swing-rate problem of the SC circuit and gives an explanation of the FAO. Section 3 presents a specific DSM circuit design scheme. Section 4 shows the test results of the chip. Finally, Section 5 provides a summary and discussion.

## 2. FIA-Assisted OTA

In a delta–sigma loop filter, the noise and linearity of the loop filter are dominated by the first-stage OTA. Achieving a low noise level and high linearity means high power consumption. SC circuits require a high slew rate (SR) for fast stabilization during switching, while the driving capability of conventional static current-biased OTAs is constrained by the current source [19]. References [5,20,21] propose active and passive charge compensation schemes, respectively. Both schemes involve adding an SC branch coupled to the output. These methods increase the drive capability of the OTA, but they also increase the power consumption and the area of the branch capacitance as well as add an extra load to the integrator inputs. The FIA offers a much larger SR during the start-up stage, without requiring current biasing. Additionally, its operating point changes with time, leading to substantially higher energy efficiency than conventional OTAs. However, the limited gain of the FIA alongside variations of operating point with PVT serve as its main constraining factors. This section begins by analyzing the effect of the SR on the integration results in the SC circuit. It then presents the design idea of the FAO to address the problem of low energy efficiency in conventional cascade circuits.

### 2.1. SR of SC Circuit

**Figure 1:**
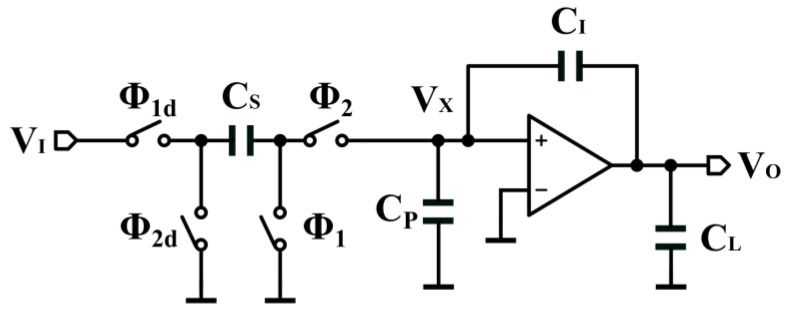
SC integrator.

The combination of gain, bandwidth, and swing rate determines the integration process in an SC integrator. Figure 1 shows the SC integrator, where $C_{P}$ is the parasitic capacitance of the OTA input and $C_{L}\;$is the load capacitance. Firstly, consider the effect of the finite gain of the OTA. The ideal OTA has infinite gain, the integrator has a pole at dc, and integration is lossless. However, the DC gain and bandwidth of OTAs are limited. Generally, $C_{P}\ll C_{S}$ and $C_{I}$. Assuming that $C_{P\;}=0$, the OTA gain is A, and the output voltage in Figure 1 reaches a value of

(1) $$V_{O}\left(\infty \right)=V_{I}\frac{C_{S}}{C_{I}}\frac{1}{1+(C_{S}/C_{I}+1)/A}$$

At the beginning of the integration phase, the sampling capacitor connects to the internal node $V_{X}$. The charge redistributes among the capacitors of the integrator, causing a voltage step at each node. Equation (2) gives the value of the voltage variation at the output:

(2) $$V_{O}\left(0+\right)=\frac{V_{I}C_{S}}{C_{S}+C_{L}(C_{S}/C_{I}+1)}$$

Interestingly, the sign for $V_{O}\left(0+\right)$ is always opposite to $V_{O}\left(\infty \right)$. As a result, the total voltage step $∆V_{O}$ is not just $V_{O}\left(\infty \right)$, but $V_{O}\left(\infty \right)+V_{O}\left(0+\right).$ Substituting into Equation (1) and Equation (2) yields:

(3) $$\Delta V_{O}=V_{I}\frac{C_{S}}{C_{I}}\left(\frac{1}{1+(C_{S}/C_{I}+1)/A}+\frac{1}{\left((C_{S}/C_{I}+1)C_{L}+C_{S}\right)/C_{I}}\right)$$

The integration process of the SC circuit can be categorized as linear, partial slewing, or totally slewing [22]. This is determined by the voltage at the input of the OTA, which results from the charge redistribution effect. Equation (4) defines the voltage at the input of the OTA:

(4) $$V_{X}\left(0+\right)=V_{I}\left(1-\frac{1}{1+C_{S}/C_{I}+C_{S}/C_{L}}\right)$$

Typically, the OTA works in the slewing region when ${|V}_{X}|>\sqrt{2}V_{\mathrm{Dsat}}$ (overdrive voltage) and otherwise in the linear region. In the slewing case, the output current of the OTA is the maximum output current I_BIAS_. When the linear condition (${|V}_{X}|<\sqrt{2}V_{\mathrm{Dsat}})$ holds, the slewing time can be expressed as

(5) $$t_{\mathrm{slew}}=\frac{\left(V_{X}\left(0+\right)-\sqrt{2}V_{\mathrm{Dsat}}\right)\left(C_{L}+C_{S}+C_{L}C_{S}/C_{I}\right)}{I_{BIAS}}$$

When the integration enters the linear region, the output current depends on the residual voltage $V_{R}=∆V_{O}-I_{\mathrm{BIAS}}t_{\mathrm{slew}}/(C_{L}+C_{S}\left|\right|C_{I})$. Assuming that the integration time is half the clock period T_S_, the final integration error ε is

(6) $$\varepsilon =V_{R}e^{-(0.5T_{S}-t_{\mathrm{slew}})/\tau },$$

where τ is given by 1/(2πGBW). Equations (3) and (6) show that $t_{\mathrm{slew}}$ must be small enough for the integrator to achieve good linearity. Therefore, the OTA needs to stabilize quickly across the slewing. However, Equation (5) shows that a small $t_{\mathrm{slew}}$ corresponds to a large I_BIAS_.

**Figure 2:**
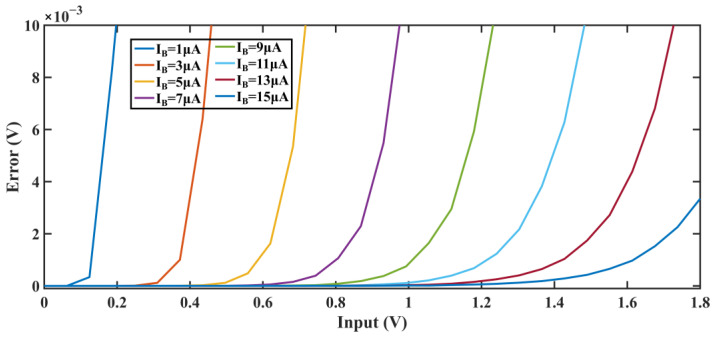
Errors vs. input voltage for various bias current.

From the previous derivation, both the load capacitance and the power of the OTA determined the integration process of the SC circuit. The capacitance value of the integrator is determined by the noise requirement and the system, making the OTA the main factor that affects integration linearity. As shown in Figure 2, assuming an OTA gain of A = 40 dB, 1/T_S_ = 6.144 MHz, C_L_ = 0.5 pF, C_S_ = 1.2 pF, C_I_ = 4.8 pF. As the current in the OTA increases, ε becomes closer to linear with respect to the input. It can be predicted that in the absence of slewing, ε is a linear function of the inputs. However, the nonlinear relationship between error and input in the existence of slewing constrains the linearity of the system. Therefore, mitigating slewing is crucial for improving linearity. During integration, the current required by the integrating capacitor decays exponentially with the growth of the output voltage. This indicates that the effect of slewing can be reduced by providing a large output current for a short time after the start of integration. The output current is then reduced to minimize power consumption. Based on this idea, the implementation is given in the next subsection.

### 2.2. Proposed FIA-Assisted OTA

**Figure 3:**
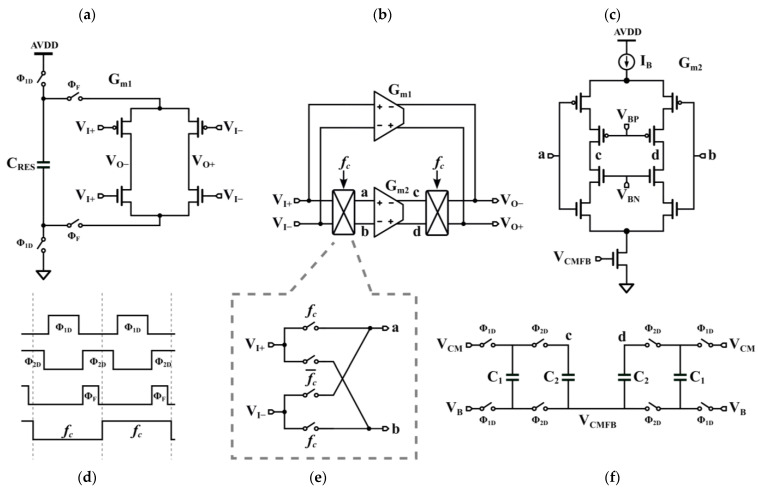
Proposed FAO: (**a**) FIA, (**b**) FAO structure, (**c**) cascode OTA, (**d**) timing diagram, (**e**) chopper and (**f**) common-mode feedback circuit.

To further mitigate the conflict between power consumption and gain, this paper presents a cascode OTA based on FIA assistance. Figure 3 shows a prototype of the FAO, wherein it is composed of a high-gain class-AB cascode OTA (Figure 3c) with an FIA (Figure 3a). The class-AB cascode OTA has a larger gm and higher current efficiency than conventional cascode structures. The reason for choosing the FIA as an assistant op-amp rather than a conventional inverter is that the FIA is more energy efficient and does not require pre-biasing. The workings of the FAO are not complicated. In the beginning of Φ_2_, the FIA connects to integrator and provides a very large current that quickly stabilizes the integrator. After a limited time, the power supply to the FIA is cut off and the cascode OTA takes over the rest of the integration. The integrator quickly enters the linear integration region with a strong drive from the FIA. The current is then supplied by the static biased OTA, resulting in acceptable linear error. Choppers (Figure 3e) at the inputs and outputs of the cascode OTA suppress flicker noise from the OTAs. They move low-frequency noise to out-of-band, thus avoiding the use of a large-sized transistor. The FIA was turned off so that it was free from noise. A conventional SC common-mode feedback circuit (Figure 3f) implements the common-mode feedback. This circuit has no limitation on the maximum output differential voltage range of the OTA and is highly linear [23].

**Figure 4:**
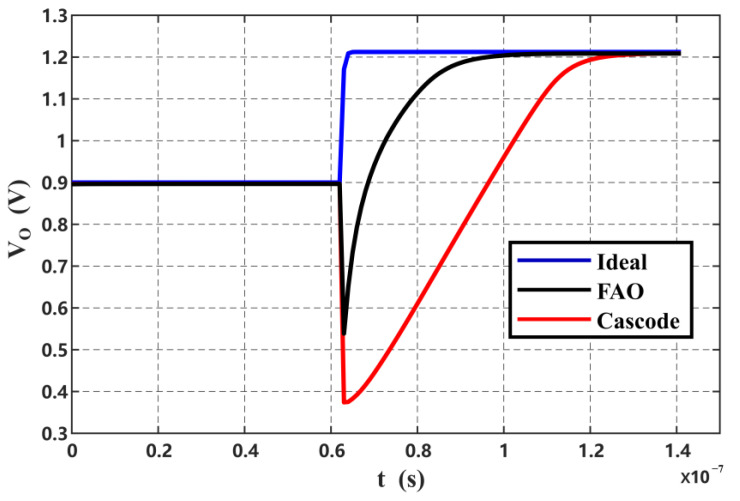
Integral curves of FAO and Cascode for the same power consumption.

The cascode OTA only ensures stable sampling of the second stage of the Φ_1_; therefore, the current is greatly reduced. In Φ_2_, large output transient currents are supplied by the FIA, which responds quickly to state switches in the integrator loop. Therefore, the low power consumption of the cascode OTA has little effect on the linearity of the output voltage. Without FIA assistance, the cascode requires approximately 40% more power than the proposed FAO for the same integration result. Figure 4 shows the step response with and without FIA assistance with the same power consumption. The cascode OTA exhibits severe slewing, while the proposed FAO settles rapidly. This advantage comes from the high current provided by the inverter structure of the FIA during the initial integration. Moreover, the FIA consumes no power after Φ_F_ is closed, and the unconsumed charge on C_RES_ can be reused in the next cycle, thus reducing the OTA’s power consumption. The closure of the FIA allows the FAO gain to be dominated by the cascode OTA, which can reach up to 80 dB.

Choosing an appropriate duty time for Φ_F_ is a worthy consideration. It must account for what happens to the circuit when the FIA turns off. When the FIA is disconnected, the charge generated by the clock feedthrough is canceled by the cascode OTA, and the parasitic capacitances of the FIA become a load on the main OTA. As previously mentioned, the main OTA has low power consumption to reduce power consumption. Thus, separating the FIA from the integrator too late or too early would increase the integration error. It is evident that disconnecting the FIA in the middle of Φ_2_ is the preferred solution. A detailed analysis will be given in the design example in the next section.

## 3. Third-Order CIFF-B Loop Filter

**Figure 5:**
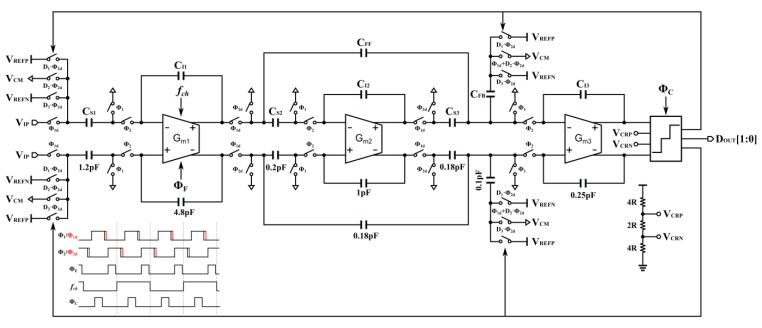
Third-order tri-level CIFF-B loop filter prototype.

Figure 5 shows the third-order tri-level cascade of integrators with a feedforward and feedback (CIFF-B) loop filter prototype. The first integrator employed the FAO, and both the second and third integrators were implemented using floating current source class-AB OTAs. The sampling capacitance of the first stage is set to 1.2 pF to achieve a dynamic range (DR) of 95 dB. The modulator can ideally achieve a signal quantization noise ratio (SQNR) of 110 dB with an out-of-band of 1.6. The quantization noise is much less than the in-band thermal noise level. This realizes thermal-noisedominated SNDR for better figures-of-merit (FoMs). The class-AB cascode structure limits the output swing of the first-stage OTA, with an output range of ±0.6 V. To suppress the back-end noise, the integration gain of the first stage should be amplified as much as possible. Using a multibit quantizer can solve this. However, the mismatch of the elements of a multibit quantizer requires an additional mismatch shaping module, which increases the power consumption and area. This design adopts a tri-level quantizer that can effectively reduce the output swing of the first integrator and results in a 6 dB reduction in the quantization noise level. The circuit can reuse the common-mode voltage (V_CM_) as the mid-level of the tri-level quantizer. Therefore, the design does not require the dynamic element matching circuitry, which is one of the major advantages of the tri-level quantizer.

### 3.1. CIFF-B Structure

The cascade of integrators with a feedforward (CIFF) structure is widely used for DTDSM. Compared with the cascade of integrators with a feedback (CIFB) structure, it reduces power consumption. Moreover, the feedforward path prevents the outputs of the first and second integrators from containing input components, reducing the output swing of the integrators. Therefore, the integrator coefficients can increase to suppress the post-stage noise. However, it is noted that the feedforward branch of the CIFF structure requires a summation operation at the input of the quantizer. The summing operation using passive adders is a simple and inexpensive scheme that is well used in single-bit quantization. Nevertheless, in multibit quantization, one has to consider the effect of kickback noise induced by the dynamic quantizer, leading to incorrect quantization results. Active adders effectively mitigate this error; the drawback is that the active adder introduces an additional offset and increases power consumption. Compared with the CIFF structure, the CIFF-B structure decouples the fast path from the exact path, thus enabling faster loop settling. Moreover, the output of the first integrator does not contain input components, inheriting the advantages of CIFF. The third-stage integrator also acts as an active adder while performing its integrating duties. The CIFF-B structure eliminates the need for an active adder at the cost of one additional digital-to-analog converter (DAC). Therefore, the CIFF-B structure is a profitable strategy.

### 3.2. First-Stage Integrator

**Figure 6:**
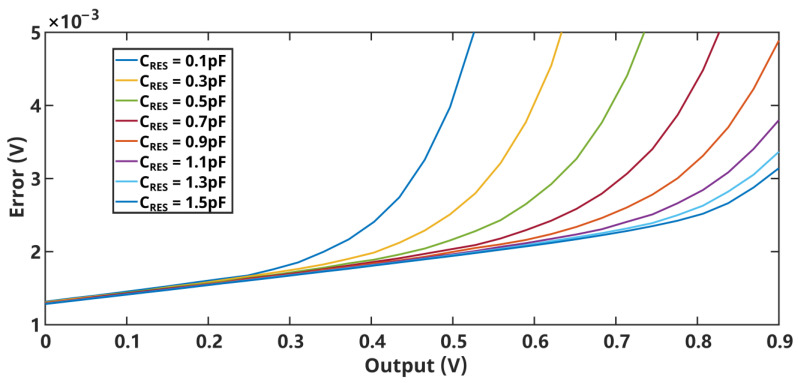
Error versus input voltage for various C_RES_.

**Figure 7:**
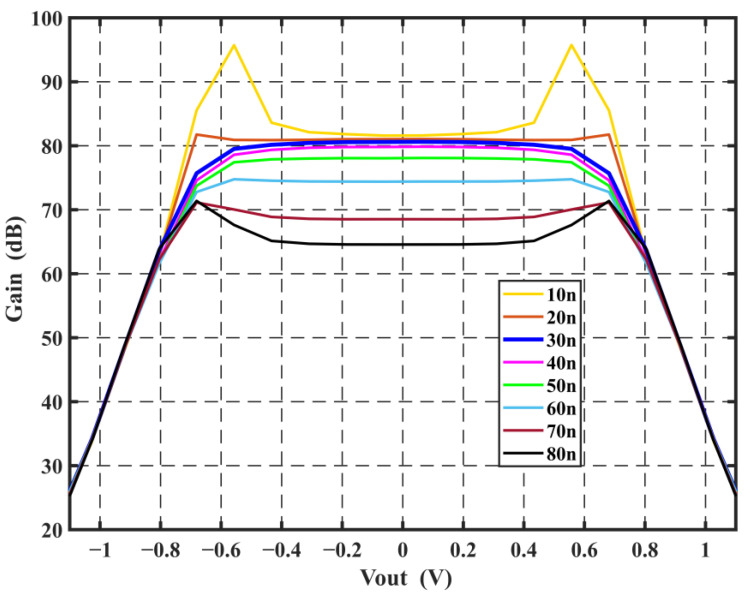
Curves of gain versus output voltage for various FIA closing times.

The input noise of the first integrator is completely restored at the output of the loop, as is the input signal of the system. Therefore, the performance of the first integrator becomes the biggest factor affecting the performance of the system. The first-stage integrator uses the FAO to solve the settling problem in Φ_2_ and decouple the back-end load from the integrator at Φ_2_ to achieve higher energy efficiency. The cascode OTA consumes 18 μA of current, considering mainly the back-end load capacitance, and has a gain of 82 dB with a unit gain bandwidth of only 13 MHz at Φ_2_. This is a far smaller bandwidth than conventional OTAs, thanks to the FIA assistance. As analyzed in the previous section, the FIA is preferably turned off in the middle of Φ_2_. Based on this setting, Figure 6 shows the simulated integral error-versus-input curve of the integrator at different C_RES_ values. The value of C_RES_ is 1.2 pF in consideration of the effect on the capacitance value by process variation. The effect of turning off the FIA at different times on the gain curve is shown in Figure 7. It can be observed that closing the FIA too early yields an unstable curve; on the other hand, closing it too late degrades the gain of the integrator. In order to prevent the PVT variation from changing the linearity of the integrator, this design reserves an adjustable range for the high level of the Φ_FIA_, which is approximately 20 to 60 ns. The FAO provides a gain of approximately 80 dB and consumes 29 μA of current, offering a low-power, high-gain solution.

### 3.3. Tri-Level Quantizer

**Figure 8:**
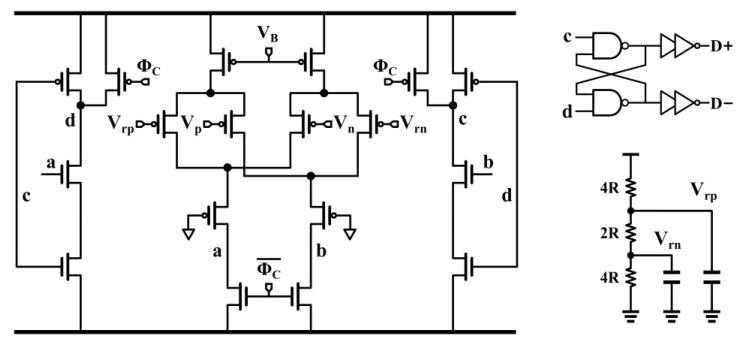
Four-input dynamic comparator unit.

The tri-level quantizer is found to be a better choice than conventional single-bit quantization. It reduces the quantization noise level, relaxes the SR of the first-stage integrator, and improves the maximum stable amplitude (MSA). A tri-level quantizer reuses the common-mode voltage present in the system as an intermediate level, and its feedback DAC has only one capacitor, like a single-bit DAC. The main advantage of a tri-level quantizer over a conventional multibit quantizer is that subsequent DACs do not require an additional mismatch shaping module, resulting in the simplification of the design. The tri-level quantizer is implemented by a 1.5-bit flash analog-to-digital converter (ADC), which contains two four-input comparators. Figure 8 shows the four-input dynamic comparator unit, which consists of a constant-current-biased preamplifier and latch. Kickback noise is further moderated by isolating the input transistor from the dynamic switch. The reference voltage of the comparator is realized by a resistor divider, and a bypass capacitor is added to suppress kickback noise. We scaled the reference voltage of the comparator to prevent the reference voltage from exceeding the comparator input range and affecting the output results.

In DSMs, the loop filter shapes the comparator offset and noise in the same way as the quantization error and has been found to have little effect on the system. However, kickback noise has been observed to swing the inputs and can cause erroneous results. Bypass capacitors at the four input terminals of the comparator can effectively reduce the kickback noise effect; increasing the power consumption of the third integrator thus appears to be necessary. A tri-level DAC uses V_CM_ as a middle level, and feedback to the input sampling capacitor yields a charge of zero. The V_CM_ not being centered on the positive and negative reference voltages produces a DAC feedback error that results in output nonlinearity. This suggests that this error can be eliminated by simply adjusting one of the reference voltages or by changing the weight of one of the output codes. The latter was chosen to be used in this design to moderate the nonlinear error by a simple off-chip calibration.

## 4. Measurement Results

**Figure 9:**
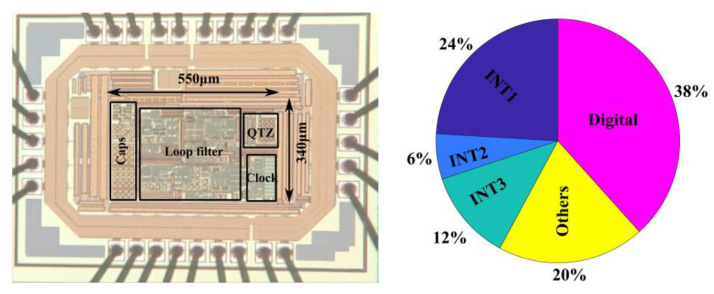
Chip micrograph and power breakdown.

The chip snapshot and power consumption distribution are shown in Figure 9. The prototype was fabricated in a 0.18 μm complementary metal-oxide semiconductor (CMOS) process with an active area of 0.19 mm^2^. An audio Precision AP-555 was used as the input source. The logic analyzer was used to enable the capture of the tri-level output of the modulator at 6.144 MS/s. The supply voltage was 1.8 V, and the power consumption was 221 μW at a sampling frequency of 6.144 MHz and a bandwidth of 24 kHz. The first integrator consumed a total of 52.2 μW of power, while the main OTAs consumed 32.4 μW. The high energy efficiency of the FIA structure makes it consume less power, while keeping integrator settling fast. In conventional designs, the power consumption of the third integrator is usually less than that of the second integrator. However, the third integrator in this design takes on the function of an active adder. In order to suppress the kickback noise of the quantizer, we increase the current of the third-stage integrator. This makes the power consumption of the third-stage integrator twice that of the second integrator.

**Figure 10:**
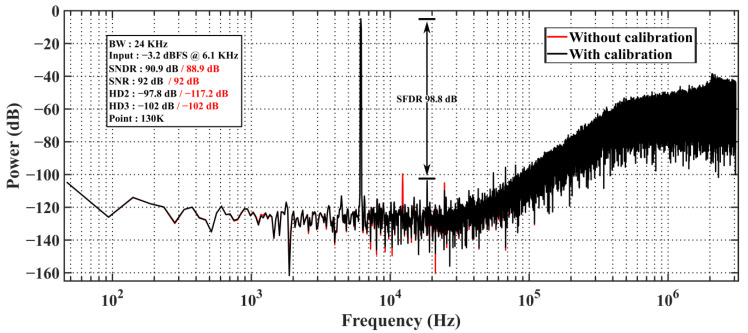
Measured output spectrum at 6.144 MHz *f_S_*.

**Figure 11:**
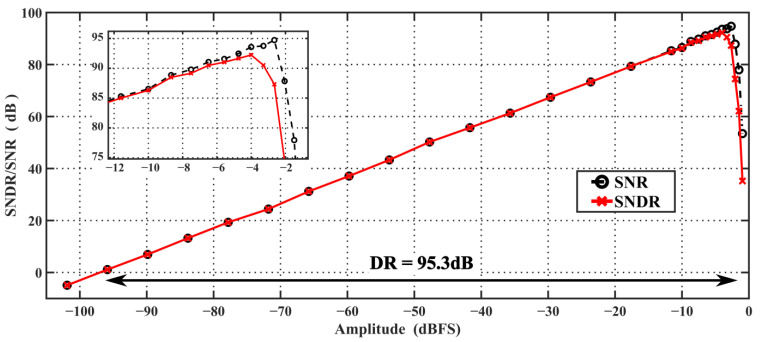
Measured SNDR/SNR versus input amplitude at 6.144 MHz *f_S_*.

Figure 10 illustrates that the input frequency of 6.1 kHz yields a peak signal-to-noise distortion ratio (SNDR)/signal to noise ratio (SNR) of 90.9/92 dB, and the spurious free dynamic range (SFDR) is 98.8 dB. The nonlinearity of the tri-level DAC, which is mainly caused by the V_CM_ not being centered on the positive and negative reference voltages, is reduced by off-chip digital calibration. Without calibration, HD_2_ is −97.8 dB, which is greater than the value of HD3 and becomes the dominant factor limiting SNDR. With digital calibration, HD2 drops to the same level as the noise floor, with negligible impact on performance. With off-chip digital calibration, the system’s SNDR peak is improved by 2 dB. Figure 11 shows the curve of SNR/SNDR as a result of the input, which was measured to be 95.3 dB for DR. The design achieves Schreier’s FoMs of FoM_SNDR_ = 171.3 dB and FoM_DR_ = 175.7 dB, respectively.

Table 1 shows a performance summary of the proposed DTSDM and a comparison with the state of the art. This design achieves comparable performance in audio bandwidth.

## 5. Conclusions

This paper presents and describes the design of a low-power, third-order DSM. An FIA-assisted OTA circuit is proposed to implement the first integrator, which inherits the advantages of both FIA and cascode OTA. The FAO achieves a high-gain, low-power integrator and improves the system’s overall energy efficiency. We showed that the operating time of the FIA in the FAO should not be too short or too long; otherwise, it will cause nonlinearity or reduce the gain. A tri-level flash quantizer was used to reduce quantization noise. The tri-level feedback DAC reuses system common-mode voltage as feedback level zero and does not require a mismatch shaping circuit. The nonlinearity caused by the mismatch of the DAC feedback voltage is eliminated by off-chip calibration, resulting in a 2 dB improvement in SNDR. The system framework uses the CIFF-B structure, which multiplexes the third integrator as an active adder to reduce the kickback effect caused by the dynamic 4-input comparator. FoM_SNDR_ = 171.3 dB and FoM_DR_ = 175.7 were obtained for the chip fabricated using the 0.18 μm CMOS process.

## Figures and Tables

**Table 1:** Performance comparison with previous work.

	This Work	[24]	[25]	[26]	[27]	[28]
Technology [nm]	180	65	180	180	65	65
Supply [V]	1.8	1	1.8	1.8	1	1
BW [kHz]	24	25	20	25	25	24
Power [μW]	220	175	103.4	68	800	94
Area [mm^2^]	0.19	0.38	1.8	0.1	0.265	0.11
SNR [dB]	92	96.1	88.7	85.1	100.1	91.9
SNDR [dB]	90.9	94.6	86.4	84	95.2	91.2
SFDR [dB]	98.8	98	91	95	-	-
DR [dB]	96.7	98.5	100	87.1	103	93
^1^ FoM_SNDR_ [dB]	171.3	176.2	169.3	169.7	170.1	175.2
^2^ FoM_DR_ [dB]	175.7	179.6	182.9	172.8	177.9	177

^1^ FoM_SNDR_ = SNDR + 10log10(BW/Power), ^2^ FoM_DR_ = DR + 10log10(BW/Power).

## Data Availability

Data available on request from the authors.
